# Retrospective analysis of pulse oximeter alarm settings in an intensive care unit patient population

**DOI:** 10.1186/s12912-016-0149-3

**Published:** 2016-06-02

**Authors:** Krystal Lansdowne, David G. Strauss, Christopher G. Scully

**Affiliations:** Office of Science and Engineering Laboratories, Center for Devices and Radiological Health, U.S. Food and Drug Administration, 10903 New Hampshire Ave, Silver Spring, MD 20993 USA

**Keywords:** Pulse oximeter, Patient monitoring, Alarm fatigue, Alarm rates

## Abstract

**Background:**

The cacophony of alerts and alarms in a hospital produced by medical devices results in alarm fatigue. The pulse oximeter is one of the most common sources of alarms. One of the ways to reduce alarm rates is to adjust alarm settings at the bedside. This study is aimed to retrospectively examine individual pulse oximeter alarm settings on alarm rates and inter- and intra- patient variability.

**Methods:**

Nine hundred sixty-two previously collected intensive care unit (ICU) patient records were obtained from the Multiparameter Intelligent Monitoring in Intensive Care II Database (Beth Israel Deaconess Medical Center, Boston, MA). Inclusion criteria included patient records that contained SpO_2_ trend data sampled at 1 Hz for at least 1 h and a matching clinical record. SpO_2_ alarm rates were simulated by applying a range of thresholds (84, 86, 88, and 90 %) and delay times (10 to 60 s) to the SpO_2_ data. Patient records with at least 12 h of SpO_2_ data were examined for the variability in alarm rate over time.

**Results:**

Decreasing SpO_2_ thresholds and increasing delay times resulted in decreased alarm rates. A limited number of patient records accounted for most alarms, and this number increased as alarm settings loosened (the top 10 % of patient records were responsible for 57.4 % of all alarms at an SpO_2_ threshold of 90 % and 15 s delay and 81.6 % at an SpO2 threshold of 84 % and 45 s delay). Alarm rates were not consistent over time for individual patients with periods of high and low alarms for all alarm settings.

**Conclusion:**

Pulse oximeter SpO_2_ alarm rates are variable between patients and over time, and the alarm rate and the extent of inter- and intra-patient variability can be affected by the alarm settings. Personalized alarm settings for a patient’s current status may help to reduce alarm fatigue for nurses.

**Electronic supplementary material:**

The online version of this article (doi:10.1186/s12912-016-0149-3) contains supplementary material, which is available to authorized users.

## Background

In acute care settings there can be as many as 350 alarms per patient per day [1]. This constant bombardment of alarms leads to ‘alarm fatigue’ causing nurse desensitization to alarms which can lead to a delayed reaction to critical events [2]. Reduction of alarm fatigue has been named a top Patient Safety Goal by the Joint Commission, as well as one of the top medical device hazards by ECRI Institute for the past few years [3, 4]. A medical device alarm should generate a response reflecting a patient’s current condition to enable timely patient assessment and, if necessary, an intervention to reverse the patient status. Multiple studies have shown that 85–99 % of alarms do not require immediate clinical intervention and occur due to mechanical issues, monitoring artifacts or the use of default alarm settings that are not adjusted to the patient [5, 6]. Exposure to non-actionable alarms has been linked to increased clinical response time to alarms [7].

One of the most common medical devices used for patient monitoring is the pulse oximeter. The pulse oximeter noninvasively and painlessly measures peripheral arterial oxygen saturation (SpO_2_) and pulse rate by a sensor that is placed on the earlobe, toe or, most commonly, the fingertip. Decreased oxygenation reflects a clinically significant event known as a hypoxemic episode (e.g., SpO_2_ less than 90 % for at least 1 min) [8]. Pulse oximeter alarms are triggered to alert nurses of a possible hypoxemic episode when SpO_2_ falls below a threshold for a pre-specified period of time (alarm delay time). These alarms can also be triggered by non-actionable or non-clinically relevant events such as patient motion or a consistently low SpO_2_ measurement that the clinical staff may already be aware of, resulting in the pulse oximeter alarm rate being one of the highest among all medical devices in many alarm studies [5, 6, 9–11]. Lawless evaluated two different pulse oximeter alarm limits and found that by relaxing the alarm settings the total amount of false alarms decreased and identification of hypoxemic episodes increased [6]. Welch found that by reducing the pulse oximeter threshold from 90 to 88 % there was a 45 % reduction in total alarms and a 70 % reduction occurred when a 15 s delay was added in a post-surgical patient population [10].

Pulse oximeters are commonly used in hospitals at their default setting. A 90 % SpO_2_ oxygen saturation is most commonly recommended as a clinical threshold for alarming a pulse oximeter during a hypoxic episode [8]. However, there is limited evidence examining alarm rates in a large number of intensive care unit (ICU) patients, and prospective randomized studies of alarm management have mostly been performed on a limited number of patients [12]. It has also been suggested that most alarms come from a limited number of patients, indicating a potential need for more personalized alarm settings. Alarm rates have been shown to vary over time and this has been attributed to numerous factors such as alarm settings, time of day, vasoactive drugs, staff to patient ratio, and pathophysiology of disease [13]. A hospital unit can define default values to be used initially for all patients, and then adjust these values as needed for an individual patient. We retrospectively examined the effect of simulated pulse oximeter alarm settings on alarm rates in an ICU patient population while considering the effect of different alarm settings on alarm rate variability between patients as well as over time for individual patients.

## Methods

### Data collection from MIMIC-II

Patient records were obtained from the Multiparameter Intelligent Monitoring in Intensive Care II (MIMIC-II) Matched Waveform Database [14, 15]. MIMIC-II Matched Waveform Database contains clinical notes, physiological monitor waveforms and vital signs from ~5,000 patient records (Waveform Database release 3) of ICU admissions collected from Beth Israel Deaconess Medical Center from 2001 to 2008. Patient records were identified that contained at least 1 h of SpO_2_ trend data record at 1 sample/s. This resulted in 962 patient records available for analysis.

### Alarm rate

From the patient data, we simulated SpO_2_ alarms at four thresholds (84, 86, 88, and 90 %) and delay times from 10 to 60 s (in increments of 5 s) to determine the expected alarm rate for each setting combination. For example, at a threshold of 90 % and delay of 15 s, we considered an alarm to be triggered for any instance where SpO_2_ was below 90 % for at least 15 s. The alarm was ‘reset’ when SpO_2_ rose above the threshold. Often for patients on continuous pulse oximetry, alarms can sound for short lengths of time due to motion artifacts (e.g., movement). To differentiate short and long periods of desaturation, we considered *corrected episodes* to be when SpO_2_ returned above the threshold within 60 s of when it initially fell below the threshold. In some patient records, SpO_2_ data were missing for lengths from a few seconds to several hours. If data was missing for less than 60 s while an alarm was being triggered and remained below the threshold when data returned, we considered the segments on either side of the missing data to represent the same alarm. If data was missing for longer than 60 s we considered separate alarms before and after the missing data. Alarm rates at four settings were examined further: 90 % threshold and 15 s delay, 88 % threshold and 25 s delay, 86 % threshold and 35 s delay, and 84 % threshold and 45 s delay. Alarm rates were assessed over all patient records as the total number of alarms in the ICU (sum of all alarms divided by sum of SpO_2_ data lengths from all patient records) and for individual patient records as discussed below.

### Interpatient variability

Interpatient variability in alarm rates, differences between patients, was assessed by considering the contribution each individual patient had to the overall alarm rate. This approach was used instead of investigating the overall number of alarms for each patient because some patients had over 100 h of SpO_2_ data recorded and other patients had only a few hours of data recorded. Additionally, patient-to-patient alarm rate differences are a factor for nurses during bedside practice when considering patient prioritization and task management. For each patient, the alarm rate was determined as the total number of simulated alarms divided by the total number of SpO_2_ h available in that patient record. The total alarm rate was defined as the sum of all individual alarm rates. The contributed alarm rate for each patient record was then their individual alarm rate divided by the total alarm rate.

### Intrapatient variability

Intrapatient variability in alarm rates, changes in alarm rate over time within individual patient records, for patient records with at least 12 h of SpO_2_ data was examined. This is important to assess over the duration of a patient stay as typically during the day shift patients undergo ordered testing, complete activities of daily living, and participate with physical and occupational therapy whereas during nights they do not often undergo testing or therapy and sleep for longer periods. For each patient record, the alarm rate was determined for all 3 h segments, if there was at least one hour of SpO_2_ data in that segment. This was used to assess the distribution of alarm rates over a patient’s ICU stay. For each patient record we determined the percentage of 3 h segments having an alarm rate greater than 1 alarm/h (i.e., if there were six 3 h segments and 2 had an alarm rate greater than 1 alarm/h, 33 % of the segments had an alarm rate greater than 1 alarm/h).

## Results

Nine hundred sixty-two patient records (514 male/444 female with 4 patient genders not reported) were identified in the MIMICII database containing at least 1 h of SpO_2_ data recorded at 1 Hz (44,900 total hours of SpO_2_ recordings, median SpO_2_ length of 29.0 h with interquartile range of 13.6 to 58.6 h). 61 patient records were noted as being greater than 90 years of age (exact ages not reported) and 4 patient ages were missing. The remaining patients average age was 63.3 ± 16 years (mean ± standard deviation). Patient records were from three intensive care units (ICU): 367 from the Coronary Care Unit (CCU), 496 from the Medical Intensive Care Unit (MICU), a unit comprising of a combination of medical/surgical patients, and 99 from the Surgical Intensive Care Unit (SICU).

### Alarm rate

**Fig. 1:**
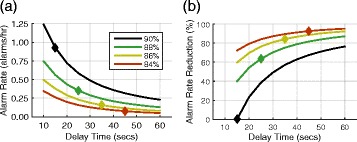
Total alarm rate and reduction. **a** Total SpO2 alarm rate over all 962 patient records for four oxygen saturation thresholds and delay times from 10 to 60 s. The diamond on each line indicates the settings detailed further. **b** Percent reduction in alarm rate caused by changing the alarm conditions relative to the alarm rate at a threshold of 90 % and delay of 15 s

As expected, SpO_2_ alarm rates estimated by combining the data from all available patient records (sum of all alarms divided by sum of all SpO_2_ data lengths) were highest at the 90 % threshold and 10 s delay setting, and alarm rates decreased when the threshold was lowered and delay time increased (Fig. 1a). At an 88 % threshold and 25 s delay there was a 62 % decrease in the alarm rate (Fig. 1b) from the 90 % threshold and 15 s delay.

At the 90 % threshold and 15 s delay setting, 70.7 % of alarm events were considered corrected episodes according to our criteria (desaturations that returned above the SpO_2_ threshold within 60 s). This was done to differentiate longer drops in SpO_2_ that could suggest deterioration in patient oxygen status from shorter episodes that may be due to artifacts (e.g., patient movement). The percentage of corrected episodes dropped to 26.7, 18.5, and 8.5 % at the alarm settings of 88 %–25 s, 86 %–35 s, and 84 %–45 s, respectively, indicating that as the alarm threshold decreased and delay time increased a higher proportion of alarms corresponded to longer desaturations indicating potentially clinically relevant hypoxemic episodes.

### Variability between patient records

**Fig. 2:**
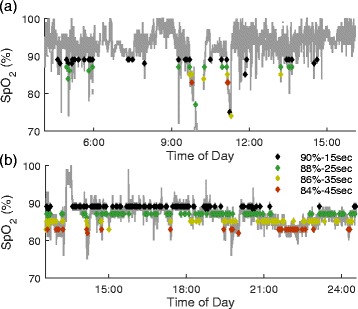
Patient record example of SpO_2_ signal and alarm thresholds. **a** Example SpO2 data from one patient during an ICU stay. Diamonds indicate time points when the alarm conditions were met (black: 90 % - 15 s, green: 88 % - 25 s, gold: 86 % - 35 s, red: 84 % - 45 s). As the threshold is decreased and delay increased, there is a reduction in the number of alarms. **b** Example SpO2 data record from another patient record. In this example, the alarm rate for each setting is high when the mean SpO2 is close to the threshold level

Figure 2 highlights the variability that can exist in alarm rates between different patients and at different SpO_2_ alarm settings. For patients with frequent short desaturations, potentially due to motion artifacts (movement of the patient), changing the thresholds can reduce the total number of alarms (see Fig. 2a). Nurses at the bedside attempt to avoid frequent alarms that result from short desaturations at the bedside by troubleshooting the SpO2 device, educating the patient on avoiding artifact producing movements as much as possible and/or lowering the SpO2 threshold of the device. For patients with an average SpO_2_ that is low and decreasing over time, changing the threshold can shift the times when alarms are frequent (see Fig. 2b).

**Fig. 3:**
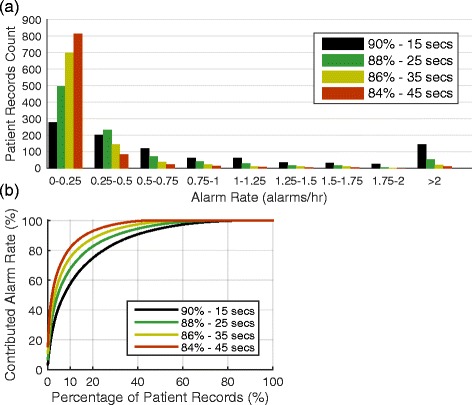
Distribution of patient alarm rates. **a** Histogram of the number of patient records at each alarm rate for four different settings. **b** The number of records accounting for the percentage of total alarm rate. For example, at a threshold of 84 % and delay of 45 s 10 % of patients account for over 80 % of all alarms

There is large variation in the alarm rate across individual patient records (Fig. 3a). Most patient records have very few alarms and as alarm settings are loosened the proportion of patient records with 0 – 0.25 alarms/h increases. A small cohort of patients was responsible for most alarms (Fig. 3b). 10 % of patient records contributed to more than 50 % of the overall alarm rate at all settings. At the alarm setting of 90 %–15 s delay, 10 % of patients contributed to 57.4 % of the overall alarm rate. These 10 % of patient records had a median alarm rate of 4.6 alarms/h. As the alarm settings loosened, the top 10 % of patient records accounted for a larger percentage of the overall alarm rate, but these patient records showed a decrease in alarms/h. When the alarm settings were set to 88 %–25 s delay, 86 %–35 s delay, and 84 %–45 s delay, the top 10 % of patient records were responsible for 67.4, 75.1, and 81.6 % of all alarms, respectively, with a median alarm rate that decreased to 2.0, 1.0 and 0.5 alarms/h.

### Variability during a patient’s ICU stay

Nine hundred fifty-two patient records had at least one 3 h segment for a total of 15,920 segments. Of these 3 h segments, 61 % had no alarms using the setting of 90 %–15 s delay. This indicates that alarm rates are not consistent over the time patients are monitored. This increased to 92 % of segments having no alarms at the setting of 84 %–45 s delay. 20.9 % of all segments had an alarm rate greater than 1 alarm/h at the setting of 90 %–15 s delay, compared with 2.2 at 84 %–45 s delay.

**Fig. 4:**
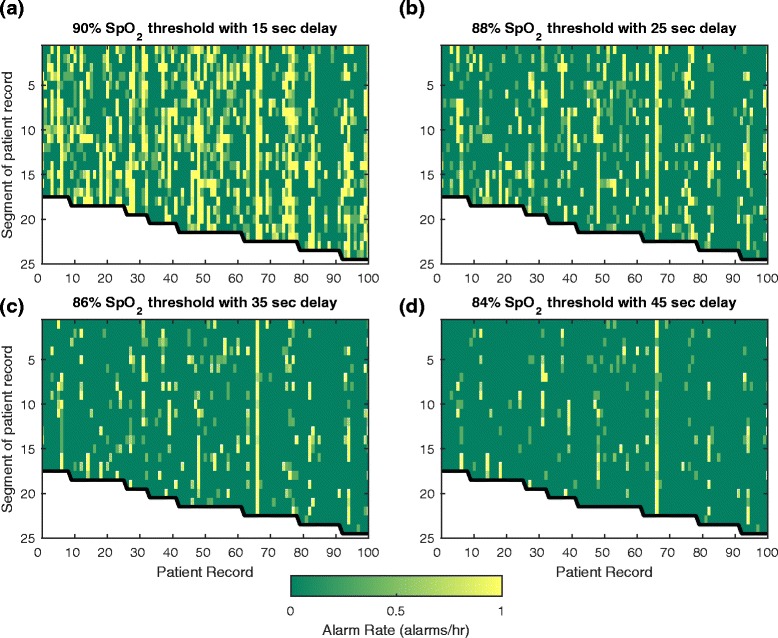
Alarm rate variability during ICU stays. Alarm rates for each 3 h segment (vertical axis) of a subset of patient records (horizontal axis) using each alarm setting: **a** 90 % SpO_2_ threshold with 15 s delay, **b** 88 % SpO_2_ threshold with 25 s delay, **c** 86 % SpO_2_ threshold with 35 s delay, and **d** 84 % SpO_2_ threshold with 45 s delay. Each column represents one patient record from the beginning of the record (top) to the end of the record (bottom) with color depicting the alarm rate over the 3 h segment, saturated at 1 alarm/h (yellow indicates alarm rate of > =1 alarm/h)

Seven hundred ninety-seven patient records had at least four 3 h segments with SpO_2_ data. Patterns of alarm rates in 3 h segments are generally inconsistent for individual patient records (Fig. 4). At settings of 90 %–15 s delay (Fig. 4a) segments with high alarm rates may be intermittent and infrequent or consistent for any individual patient record. At 84 %–45 s delay (Fig. 4b) periods of high alarm rates are isolated except for a single patient record that has consistently high alarm rates. For visualization purposes only a subset of patient record alarm rate patterns are shown in Fig. 4. Alarm rate patterns for all patients with at least four 3 h segments are available in Additional file 1.

**Fig. 5:**
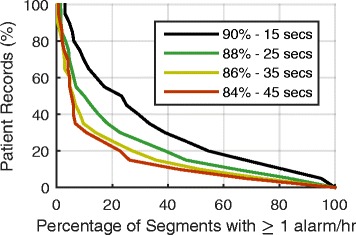
Consistency of high alarm rates. The percentage of patient records having X percent of 3 h segments with alarm rates ≥ 1 alarm/h, for patient records with a minimum of 12 h of SpO_2_ data. For example, 55 % of patient records have at least 20 % of segments with an alarm rate of ≥ 1 alarm/h at the alarm setting of 90 %–15s. At 84 %–45s only 23 % of all records have at least 20 % of segments with an alarm rate of ≥ 1 alarm/h

We identified patient records with at least one 3 h segment having an alarm rate ≥ 1 alarm/h. For the four settings there were 565, 372, 249, and 149 patient records (90 %–15 s delay to 84 %–45 s delay) with at least one 3 h segment having an alarm rate ≥ 1 alarm/h. Few patients have a large proportion of segments with high alarm rates (Fig. 5) indicating that for most patients high alarm rates are not consistent throughout an ICU stay. At a setting of 90 %–15 s, ~55 % of the 565 patient records (those with at least one 3 h segment with a high alarm rate) have an alarm rate greater than 1 alarm/h in at least 20 % of segments. This indicates that close to half of all patient records (~45 %) with a high alarm rate in at least one 3 h segment did not have a high alarm rate throughout their ICU stay (more than 80 % of all the 3 h segments have less than 1 alarm/h). For a setting of 84 %–45 s, only 23 % of patient records had an alarm rate ≥ 1 alarm/h in more than 20 % of segments.

## Discussion

The number of SpO_2_ alarms (both true-positive and false-positive) is related to the alarm settings and a patient’s current condition. Patient monitor threshold alarms, such as examined in this study, can provide an indication to the caregiver that there is a potential acute change in a patient’s status requiring reassessment. However, frequent alarms do not always correspond to such need for action. By modifying the alarm settings in an intensive care unit patient population, the overall alarm rate significantly decreased (62 % decrease when alarm setting changed from 90 % SpO_2_ threshold with 15 s delay to 88 % SpO_2_ threshold with 25 s delay). By retrospectively examining the varying alarm rates of the pulse oximeter, it was shown that most of the alarms came from a limited number of patients (10 % of patients were responsible for more than half of all alarms at any setting). The small cohort of patients accounting for most of the alarms and the variability in alarm rates in ICU stays suggests a need for personalized alarm settings.

Numerous studies have found that by adjusting the threshold and delay time on the pulse oximeter, the alarm rate decreased almost by half or more [5, 6, 9–11]. The decrease in alarm rate is consistent with a prior study in which alarm settings were changed on a hospital unit and reduced the critical monitoring alarms, including those from the pulse oximeter, by 43 % [16]. However, continuing to lower the threshold and increase the delay time may reduce the alarm rate with diminishing returns and could also increase the response time to a clinically relevant event. Such adjustments have to be balanced against the need for early notification of clinically relevant events. The nurse at the bedside is able to do certain things to mitigate the alarm load, to include suspension of alarms for a short period before directly interacting with the patient, documenting alarm parameters in patients’ medical record, and adjusting alarms to a patient’s actual needs [16]. However, nurse workload, improved physiological monitoring algorithms, alarm regeneration, hospital noise reduction strategies, and actionable alarm limits designated by the hospital all play a role to reduce alarms [16, 17].

A small number of patients accounted for most of the overall alarm rate. As the alarm settings changed, there was an increase in the contribution of the top 10 % of patients to the overall alarm rate as the total number of alarms decreased. For example, at 84 %–45 s delay, the top 10 % of patients were responsible for more than 80 % of the overall alarm rate. This small group of patients may be candidates for more individualized alarm management, if reducing the high frequency of alarms does not affect the quality of care. Further research is needed to identify specific patient populations/units and factors, (e.g., patient primary diagnosis, isolation, lab values, comorbidities, history and lifestyle) that can guide personalized alarm settings. The bedside nurse is often the first to gather the patients’ baseline physiological data during assessment in an ICU. This assessment information obtained by the nurse may allow for a proactive adjustment to the alarm setting, especially if the patients’ baseline SpO_2_ is normally below the default setting, which is commonly found in respiratory failure, systemic inflammatory response syndrome or chronic obstructive pulmonary disease. The pulse oximeter can measure how well the hemoglobin is being saturated, but it cannot discern what exactly it is being saturated with, which can lead to false readings (e.g., saturated with oxyhemoglobin or carboxyhemoglobin in carbon monoxide poisoning) [18]. Individualized pulse oximeter alarm settings may allow nurses to still be readily notified of critical events while reducing overall alarm rates.

Our analysis of the variability within a patient’s ICU stay suggests that the alarm settings need to not only be set for individual patients but also adjusted throughout an individual’s stay, as very few patients had consistently high alarm rates. This alarm variability over time is indicative of the rapidly changing nature of a patient and how adjustment of settings and assessment of the patient may be needed. Although individual alarms during high alarm rate periods may be a nuisance, the periods themselves may be clinically relevant to the bedside nurse. As a result, the patient and the alarm settings together affect alarm consistency and variability over the ICU stay. Nurses assess a patient consistently while under their direct care. The patients’ status may change suddenly for the better or worse, triggering the nurse to administer or stop medication, provide non-pharmacological interventions, and/or adjust alarm settings. A deteriorating patient may result in a lower mean SpO_2_ that fluctuates around the alarm set point, repeatedly triggering alarms. An improving patient may begin moving around more, or a deteriorating patient may receive more clinical interventions resulting in more motion artifacts and artificial drops in SpO_2_. Our analysis indicates that alarms are not consistent throughout an ICU stay, and there are often periods with high alarm rates and periods of no alarms. These periods of high alarm rates can be due to motion artifacts from clinical interventions (procedures, labs etc.) or the patient themselves, or a sign that the patient status is changing. Nurses however, do not assess a patients’ status based on one physiological signal. Nurses are trained to perform a global assessment of the patient taking information from multiple factors. For example, a patient with advanced emphysema on 3 l of oxygen and a saturation of 93 % would not be considered a clinical emergency. However, a patient with an SpO_2_ of 93 % along with altered mental status, tachycardia and cyanosis of oral mucous membranes or skin would be considered an emergency requiring an intervention [19]. However these physical changes of low oxygen saturation are subtle and can be difficult to discern. This ability to do a global assessment may be best mimicked by the development of improved physiological monitoring devices with intelligent alarm systems that take into account other physiological parameters from multiple signals or medical devices to determine if an alarm is clinically relevant [20].

This study was performed as a retrospective analysis of a previously collected database and therefore has limitations. We could not determine if each individual desaturation was actually a ‘clinically relevant’ event. In addition, if a clinical intervention did occur due to an alarm that was triggered at the bedside monitor settings (e.g., 90 % threshold with 10 s delay) we do not know if the lower alarm settings would have been met as well or what the delay in response would have been from these lower settings.

Numerous studies have shown that the different algorithms used by manufacturers in a pulse oximeter device can affect their accuracy [13]. Pulse-oximeter devices may also have improved their signal processing since the time this data was collected (2001 to 2008) as well as different patient clinical monitoring protocols may have been adopted since the time the data was collected. We therefore simply treated each signal as a continuous SpO_2_ trend. It is important to consider that responding to a desaturation and applying a clinical intervention (or observing that an intervention is not necessary) at one event may affect the clinical response in future events. We also did not consider a post-alarm refractory period, which is whether SpO_2_ had to stay above the threshold for a certain amount of time before dropping below the threshold again to trigger a new alarm. For these reasons the alarm rates presented here may not represent those achieved by prospectively setting these alarm conditions and monitoring alarm rates in a modern intensive care unit. Given these limitations, we believe that this study design allowed a unique examination of the effect of changing alarm conditions on alarm rates and patterns in a large (962 patient records, 44,900 h of SpO_2_ data) ICU patient population that may present considerations for future alarm fatigue study designs.

## Conclusion

Intensive care unit patients are some of the most vulnerable and critically ill patients. They require continuous monitoring from an assortment of medical devices, with the pulse oximeter being just one of them. Devices ideally should provide an early notification of a change in patient status to nurses. Unfortunately, medical devices are not without their errors and can trigger alarms repeatedly for non-clinically relevant events. This ‘noise’ results in alarm fatigue for the nurse whose critical thinking skills have to consistently identify and, if needed, intervene during an actual clinically relevant event. By adjusting alarm thresholds and delays the ‘noise’ of non-clinically relevant alarms may be reduced. This decrease in the overall alarm rate may result in a reduction in alarm fatigue and potentially an improved response to clinically relevant alarms; however, assessing the potential risk to missing or delaying the response to true alarms by increasing the delay time or decrease the setting was beyond the scope of what could be assessed in this study. This will depend on the condition of the particular patient and highlights the potential importance of individualizing alarm settings. We have shown that alarm rates are not constant between patients or within an individual’s ICU stay. Further research is needed to identify for which patients and when alarm settings should be adjusted.

## Additional file

Additional file 1: Presents the alarm rates throughout the full patient records at each of the four alarm settings for all patient records with at least four 3 hour segments. (DOC 208 kb)

## Abbreviations

CCU: coronary care unit
ICU: intensive care unit
MICU: medical intensive care unit
MIMIC II: multiparameter intelligent monitoring in intensive care II
SICU: surgical intensive care unit
SpO2: peripheral oxygen saturation
