# Supplementary material for: Retrospective analysis of pulse oximeter alarm settings in an intensive care unit patient population
Source: BMC Nurs. 2016 Jun 2;15:36. doi: 10.1186/s12912-016-0149-3 (PMC4891882; doi:10.1186/s12912-016-0149-3)

**Supplementary Information**

Alarm rates over time for patient records with at least four 3 hour segments at each setting. Horizontal axis indicates patient record sorted by record length. On the vertical axis, from top to bottom each grid point indicates adjacent 3 hour segments from the start to end of each patient record. Color indicates alarm rate from 0 to ≥1 alarms/hr.


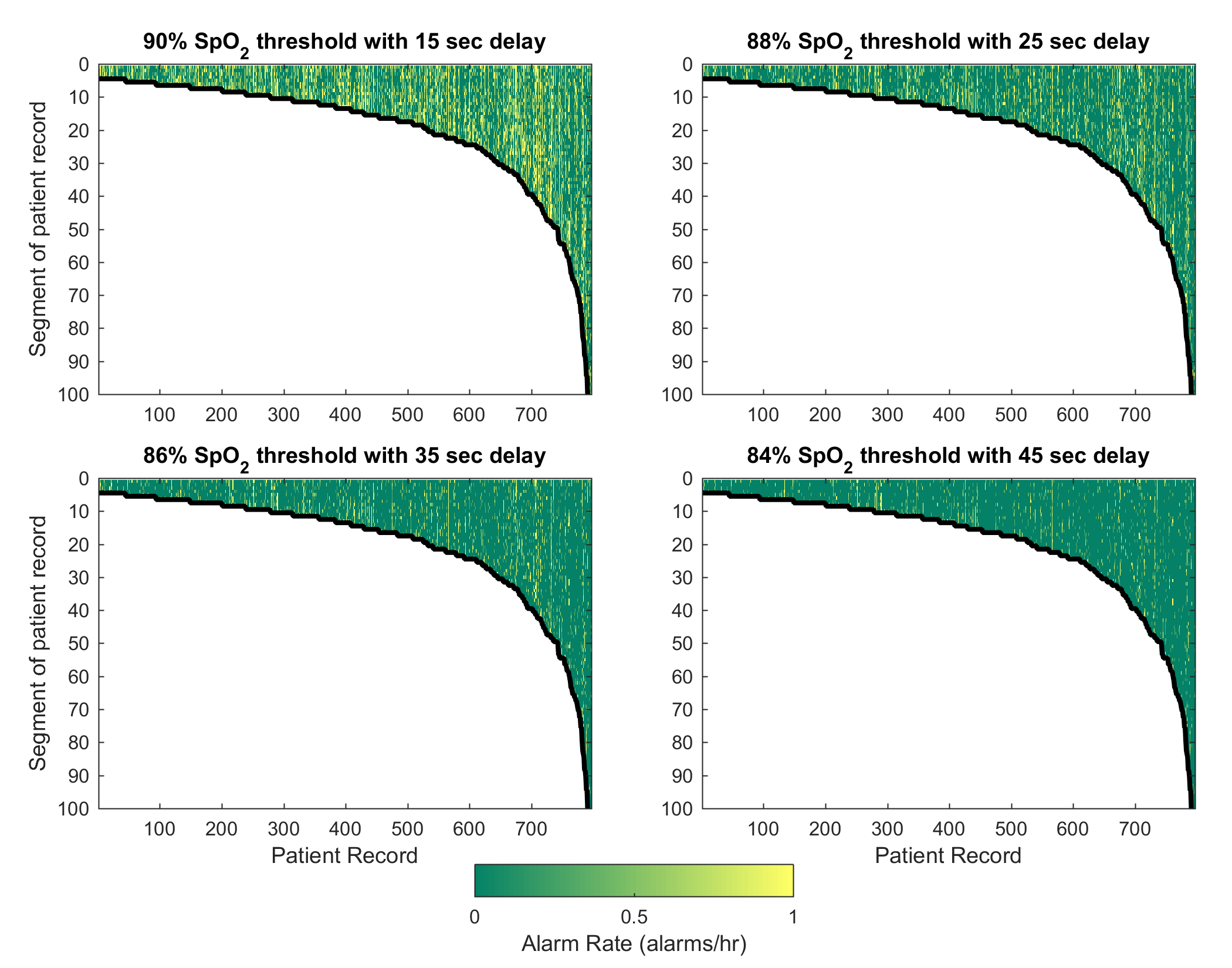

Supplement: Additional file 1: — Presents the alarm rates throughout the full patient records at each of the four alarm settings for all patient records with at least four 3 hour segments. (DOC 208 kb) [file 12912_2016_149_MOESM1_ESM.doc]
